# A 2-year cohort study of ocular bioparameters in non-myopic primary schoolchildren with different refractive states

**DOI:** 10.1097/MD.0000000000047486

**Published:** 2026-01-30

**Authors:** Chengcheng Han, Rui Zhou, Yuyang Wu, Haiyan Ma, Qingjie Meng, Zhiping Zhang

**Affiliations:** a Optometry Center, Heilongjiang Provincial Eye Disease Control Centre/Heilongjiang Provincial Eye Hospital, Harbin, China.

**Keywords:** axial elongation, hyperopic reserve insufficiency, incident myopia, pre-myopia, refractive error progression

## Abstract

This study aimed to investigate the determinants of early myopic progression in emmetropic children through a comprehensive analysis of refractive profiles, axial length (AL), and axial length/corneal radius (AL/CR) ratio. A cohort study was conducted on 206 non-myopic primary school students (age: 6–10 years). Participants were stratified into 3 hyperopic reserve groups: adequate, relatively insufficient, and severely depleted. longitudinal monitoring over 24 months included annual cycloplegic refraction measurements, AL, and AL/CR ratio. At the 12-month follow-up, the incidence of myopia was 32.5%. The 3 groups exhibited statistically significant differences in spherical equivalent progression (−0.70 ± 0.46 D vs −0.56 ± 0.45 D vs −0.38 ± 0.36 D; *P* < .001) and AL elongation (0.40 ± 0.17 mm vs 0.31 ± 0.18 mm vs 0.27 ± 0.16 mm; *P* < .001). At the 24-month follow-up, the cumulative incidence of myopia was 52.4%. The 3 groups exhibited significant differences in spherical equivalent progression (−1.31 ± 0.67 D vs −1.02 ± 0.68 D vs −0.86 ± 0.65 D; *P* < .001) and AL elongation (0.74 ± 0.29 mm vs 0.59 ± 0.26 mm vs 0.56 ± 0.31 mm; *P* < .001). Baseline AL/CR ratios differed significantly across groups (3.00 ± 0.50 vs 2.94 ± 0.49 vs 2.87 ± 0.63; *P* < .001). Multivariate logistic regression analysis confirmed relative hyperopic insufficiency as an independent risk factor for myopia onset over 24 months. The phase of suboptimal hyperopic reserve serves as a precursor biomarker for pre-myopia, warranting prioritized monitoring for proactive myopia control. To optimize preventive efficacy, targeted interventions should be initiated during the second annual follow-up in longitudinal cohorts.

## 1. Introduction

With the global prevalence of myopia and high myopia steadily increasing,^[1]^ myopia prevention and control have become crucial public health issues. Although reducing the progression rate of myopia remains a priority in current research, preventing its onset is a more valuable objective. Preventing insufficient hyperopic reserves in children and their premature transition to the emmetropic stage can effectively reduce the occurrence and development of myopia.^[2]^ Therefore, research on hyperopic reserves and their practical applications is of significant importance.

The emmetropic concept proposed by the International Myopia Institute defines a refractive state in children as a spherical equivalent (SE) of ≤+0.75 and >−0.50 D. Subsequent updates in the 2022 Asian Consensus on Myopia Management and the White Paper on Myopia Management introduced age-stratified refinements to the pre-myopia SE thresholds^[3,4]^: +0.75 D ≥ SE > −0.50 D for 6-year-olds, +0.50 D ≥ SE > −0.50 D for 7 to 8-year-olds, +0.25 D ≥ SE > −0.50 D for 9 to 10-year-olds, and +0.00 D ≥ SE > −0.50 D for 11 to 12-year-olds. The diagnosis incorporates baseline refraction, age, and quantifiable risk factors.^[5]^ Despite its clinical significance, a consensus regarding the precise definition of emmetropia remains elusive.^[6]^ Emerging evidence has identified hyperopic reserve as a practical predictor of myopia onset, with insufficient hyperopic reserve intrinsically linked to pre-myopia.^[7-10]^ The concept of hyperopic reserve originates from the understanding of the characteristic changes in refractive development during childhood and adolescence. Most newborns are born with hyperopic eyes, followed by continuous axial elongation that gradually reduces hyperopia and approaches emmetropia, a process known as emmetropization.^[11]^ The magnitude of hyperopia present during this process is referred to as hyperopic reserve.^[12]^ This measure helps differentiate between rapid and stable trajectories of myopia progression.^[13]^ A longitudinal investigation of the transition from suboptimal hyperopic reserve to established myopia may elucidate dynamic patterns of refractive development.

This longitudinal cohort study enrolled 206 emmetropic primary school students who underwent baseline and 12 and 24-month follow-up examinations. Cycloplegic refraction, axial length (AL), and axial length/corneal radius (AL/CR) ratio measurements were systematically performed to quantify the incidence of myopia onset, characterize the refractive dynamics and biometric alterations, and provide an evidence-based framework for preventive myopia control strategies.

## 2. Materials and methods

### 2.1. Study design and sample

This retrospective cohort study enrolled 220 school-aged children (aged 6–10 years) who underwent baseline examinations between January 2022 and December 2022. Participants were recruited from patients receiving care at the Optometre Hospital, Heilongjiang Provincial Eye Hospital. The study adhered to the tenets of the Declaration of Helsinki and received ethical approval from the Institutional Review Board of Heilongjiang Eye Hospital, Chinay Clinic. Of these, 206 primary school students completed the study. Inclusion criteria comprised: school-aged children (6–10 years); cycloplegic spherical equivalent refraction between −0.25 and +3.00 D; astigmatism ≤ 1.50 D; anisometropia ≤ 1.00 D; uncorrected visual acuity ≥ 0.10 log MAR in both eyes; and no prior myopia control interventions. The exclusion criteria were systemic comorbidities, ocular pathologies, or a history of pharmacological or optical myopia control.

At baseline, ophthalmic evaluations included AL, the AL/CR ratio, and cycloplegic objective refraction. Follow-up assessments were performed at 12 and 24 months after enrollment. Uncorrected visual acuity was evaluated using a standard 100% contrast tumbling E Log MAR chart at 5 m. Objective refraction was performed under cycloplegia using an autorefractor (NIDEK ARK-1, Japan). Four repeated measurements were obtained for each eye, and the average spherical equivalent refraction was recorded for data analysis. Cycloplegia was achieved through sequential instillation of 4 drops of 1% cyclopentolate hydrochloride (Alcon Laboratories, Fort Worth) at 5-minutes intervals. Accommodation was assessed 30 minutes post-instillation using an autorefractor (NIDEK ARK-1, Japan). If the residual accommodation exceeded + 2.00 D, additional cycloplegic drops were administered. Once cycloplegia was confirmed, an objective refraction was performed. Optical biometry (IOLMaster 500, Carl Zeiss Meditec, Jena, Germany) was used to obtain 5 consecutive measurements of the AL and corneal radius, with the mean value used for analysis. The SE was calculated as a sphere +½ cylinder from the baseline cycloplegic measurements. Myopia progression was defined as SE ≤ −0.50 D under cycloplegia at annual follow-ups.

### 2.2. Grouping and data processing

Non-myopic schoolchildren were stratified into 3 cohorts based on baseline cycloplegic SE measurements. Stratification criteria were established using the pre-myopia diagnostic thresholds outlined in the Myopia Management White Paper (2022) and Asian Consensus on Myopia Control; age-specific normative values for hyperopic reserve were derived from the Chinese Expert Consensus on Ocular Biometric References (incorporating AL, corneal curvature, and genetic predisposition metrics).^[3,4,14]^ The cohorts included – Adequate hyperopic reserve group: meeting age-specific reference values per the Chinese Expert Consensus on Ocular Biometric Norms; Relative hyperopic insufficiency group: hyperopia reserve above the lower limit but below the recommended threshold; and Severely insufficient hyperopic reserve group (pre-myopia group), classified as pre-myopia under the 2022 Myopia Management White Paper and Asian Myopia Consensus criteria.

### 2.3. Statistical analyses

Statistical analyses were performed using SPSS version 26.0 (SPSS Inc., Chicago). The right-eye data were used for analysis and reporting. Continuous variables were compared using analysis of variance and categorical variables were compared using the chi-square test. Differences were considered statistically significant at *P* ≤ .05. Multivariate analysis was performed using logistic regression analysis.

## 3. Results

In the non-myopic cohort (n = 206), 67 participants (32.5%) developed myopia during the initial 12-month follow-up period. A hierarchical risk gradient was observed across the hyperopic reserve groups: the severely depleted hyperopic reserve group demonstrated the highest conversion rate, followed by the relative hyperopic insufficiency group, with the adequate hyperopic reserve group exhibiting the lowest incidence (*P* < .001). At 24 months, the cumulative incidence of myopia increased to 52.4% (108/206), maintaining significant intergroup differences (Severe Depletion > Relative Insufficiency > Adequate Reserve; *P* < .001; Table 1).

**Table 1:** Refractive status at 12 and 24 months in 3 groups.

Groups		Severely insufficient hyperopic reserve group	Relatively insufficient hyperopic reserve group	Sufficient hyperopic reserve group	n	χ^2^	*P*
12 mo	Myopic	63 (65.6%)	4 (6.7%)	0	67 (32.5%)	201.104	<0.001
	Severe depletion	33 (34.3%)	29 (48.3%)	1 (2.0%)	63 (30.6%)		
	Relative insufficiency	0	27 (45.0%)	19 (38.0%)	46 (22.3%)		
	Adequate reserve	0	0	30 (60.0%)	30 (14.7%)		
24 mo	Myopic	85 (88.5%)	21 (35.0%)	2 (4.0%)	108 (52.4%)	177.840	<0.001
	Severe depletion	11 (11.5%)	26 (43.3%)	9 (18.0%)	46 (22.3%)		
	Relative insufficiency	0	13 (21.7%)	14 (28.0%)	27 (13.1%)		
	Adequate reserve	0	0	25 (50%)	25 (12.1%)		
Total		96	60	50	206		

**Figure 1.**
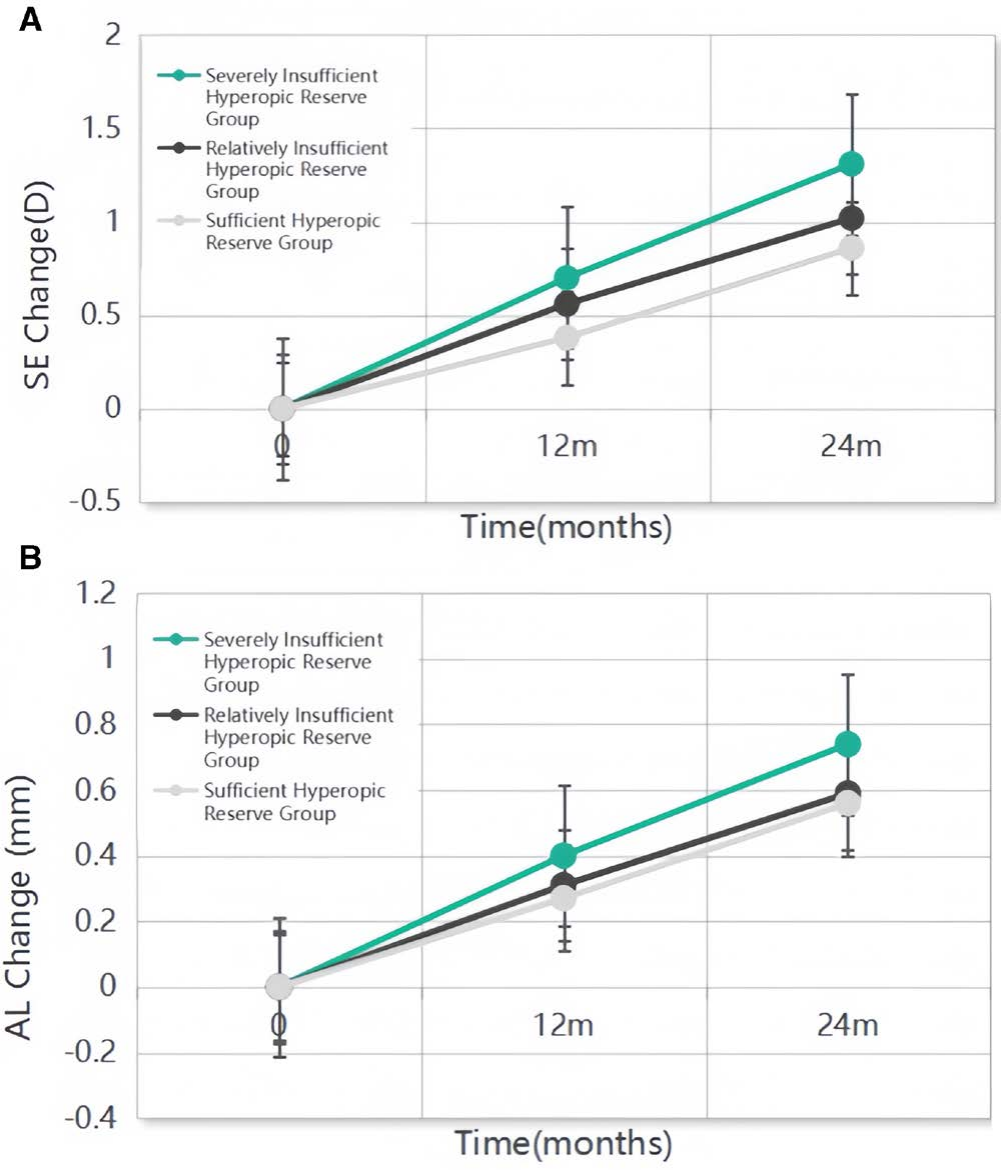
Comparison of SE and AL growth at 12 and 24 months among the 3 groups. (A) Comparison of SE growth at 12 and 24 months among the 3 groups. (B) Comparison of SE and AL growth at 12 and 24 months among the 3 groups. The data are expressed as the mean and standard error of the mean. AL = axial length, SE = spherical equivalent.

At 12 months, the changes in SE and AL differed significantly among the severely depleted hyperopic reserve, relative hyperopic insufficiency, and adequate hyperopic reserve groups. SE progression was −0.70 ± 0.46 D, −0.56 ± 0.45 D, and − 0.38 ± 0.36 D, respectively (*F*_2, 203_ = 8.878, *P* < .001). Corresponding AL elongation measured 0.40 ± 0.17 mm, 0.31 ± 0.18 mm, and 0.27 ± 0.16 mm (*F*_2, 203_ = 9.423, *P* < .001). A dose-dependent relationship was observed, with each 1 mm increase in AL associated with SE progression of −1.75, −1.80, and −1.40 D in the severely depleted, relative insufficiency, and adequate reserve groups, respectively. At 24 months, 23 participants who developed myopia at 12 months received myopia control intervention with defocus-incorporated multiple segments (DIMS) spectacles, while the remaining 183 children remained untreated. Among untreated participants, SE progression reached −1.31 ± 0.67 D, −1.02 ± 0.68 D, and −0.86 ± 0.65 D across the 3 reserve groups (*F*_2, 180_ = 7.229, *P* < .001), with corresponding AL elongation of 0.74 ± 0.29 mm, 0.59 ± 0.26 mm, and 0.56 ± 0.31 mm (*F*_2, 180_ = 7.100, *P* = .001; Fig. 1).

Significant disparities in refractive and biometric table progression were observed between myopic converters and non-converters during the 12-month follow-up. The myopic conversion group exhibited SE progression of −0.95 ± 0.46 D compared to − 0.41 ± 0.32 D in non-converters, demonstrating significant differences (*F*_1, 204_ = 95.168, *P* < .001). AL elongation was similarly greater in converters than in non-converters (0.47 ± 0.19 mm vs 0.28 ± 0.14 mm; *F*_1, 204_ = 61.286, *P* < .001; Table 2).

**Table 2:** Comparison of SE and AL growth in myopic and non-myopic eyes at 12 months.

Group	The myopia-developed group	The non-myopia-developed group	*F*-value	*P*-value
Spherical equivalent (D)	−0.95 ± 0.46	−0.41 ± 0.32	95.168	<.001
Axial length (mm)	0.47 ± 0.19	0.28 ± 0.14	61.286	<.001

AL = axial length, SE = spherical equivalent.

Among the 67 myopic converters, 23 received DIMS spectacle interventions, while 44 wore single-vision lenses. The remaining 139 non-converters did not receive pharmacological or optical control of myopia. Second-year progression analysis revealed distinct efficacy profiles: SE of −0.78 ± 0.42 D and AL of 0.42 ± 0.21 mm for single-vision lenses wearers, SE of −0.32 ± 0.18 D and AL of 0.15 ± 0.10 mm for DIMS wearers, and SE of −0.49 ± 0.40 D and AL of 0.29 ± 0.19 mm for non-converters. Intergroup comparisons demonstrated significant differences in SE (*F*_2, 203_ = 13.625, *P* < .001) and AL (*F*_2, 203_ = 17.170, *P* < .001; Table 3).

**Table 3:** Comparison of SE and AL growth in year 2 between different modes of myopia correction occurring within 12 months.

Group	The myopia-developed group		The non-myopia-developed group	*F*-value	*P*-value
	SVL	DIMS			
Spherical equivalent (D)	−0.78 ± 0.42	−0.32 ± 0.18	−0.49 ± 0.40	13.625	<.001
Axial length (mm)	0.42 ± 0.21	0.15 ± 0.10	0.29 ± 0.19	17.170	<.001

DIMS = defocus-incorporated multiple segments, SE = spherical equivalent, SVL: single-vision lenses.

The severely depleted hyperopic reserve group exhibited significantly higher AL/CR ratios than the other cohorts (*F*_2, 203_ = 91.202, *P* < .001). Children who developed myopia within the 24-month follow-up demonstrated higher baseline AL/CR values (3.00 ± 0.53) than those maintaining non-myopic status (2.91 ± 0.63), with significant intergroup differences (*F*_1, 204_ = 109.98, *P* < .001; Table 4).

**Table 4:** Comparison of AL/CR between the groups.

Group	AL/CR	*F*-value	*P*-value
Grouped according to baseline SE		91.202	<.001
Severely insufficient hyperopic reserve group	3.00 ± 0.50		
Relatively insufficient hyperopic reserve group	2.94 ± 0.49		
Sufficient hyperopic reserve group	2.87 ± 0.63		
Myopia occurs after 2 yr or not		109.98	<.001
Myopia	3.00 ± 0.53		
Non-myopia	2.91 ± 0.63		

AL/CR = adaxial length/corneal radius, SE = spherical equivalent.

Multivariate logistic regression analysis was performed to evaluate the risk factors associated with 24-month myopia incidence among non-myopic schoolchildren (23 children receiving DIMS lenses were excluded from the final regression model), with myopia onset (yes/no) as the dependent variable. The analysis identified severely depleted hyperopic reserves and relative hyperopic insufficiency as significant independent predictors of myopia development (*P* < .001; Table 5).

**Table 5:** Multifactorial logistic regression analysis associated with the occurrence of myopia in primary school children at 24 months.

Independent variable	*B*	Standard error	Wald χ^2^-value	*P*-value	OR-value (95%)
Age	−0.034	0.164	0.042	.837	0.967 (0.701–1.333)
Sex (man)	0.250	0.404	0.384	.535	1.285 (0.582–2.835)
Insufficient hyperopic reserve			63.659		
	5.275	0.798	43.721	<.001	195.395 (40.912–933.211)
	2.580	0.775	11.083	<.001	13.200 (2.890–60.294)
Severely insufficient hyperopic reserve	−0.034	0.164	0.042	.837	0.967 (0.701–1.333)
Relatively insufficient hyperopic reserve	0.250	0.404	0.384	.535	1.285 (0.582–2.835)

OR = odds ratio.

## 4. Discussion

Substantial heterogeneity exists in the operational definitions of myopia across the studies. Some researchers define this condition as an annual myopic progression exceeding −0.50 D in children with baseline SE below + 1.00 D. In contrast, others define it as static refractive thresholds ranging from +1.00 to −1.50 D.^[15-17]^ This persistent definitional ambiguity underscores the need for comprehensive research to establish biologically validated diagnostic criteria that clarify its pathophysiological determinants and enable reliable differentiation between schoolchildren with pre-myopia and their stable emmetropic peers.

During the 24-month follow-up in this cohort study, 32.5% of schoolchildren developed myopia in year 1, with a cumulative incidence of 52.4% by year 2, consistent with previous epidemiological benchmarks.^[15,18]^ The Shanghai Epidemiologic Study on Pre-Myopia Transition reported comparable progression rates, with 31.5% annual conversion and 62.0% 2-year cumulative incidence among children with pre-myopia, substantially higher than those in hyperopic controls.^[19]^ In the present study, the severely depleted hyperopic reserve group (pre-myopia cohort) demonstrated accelerated progression with 65.6% first-year and 88.5% cumulative second-year incidences. These higher rates may be attributed to variations in age according to the inclusion criteria and stratification protocols. These findings highlight the persistently high clinical conversion rates from pre-myopia to myopia and the limitations of current prevention strategies. While established interventions, including low-dose atropine, orthokeratology, defocus-incorporated spectacles, and multifocal soft contact lenses, demonstrate efficacy in children with myopia,^[20,21]^ preventive measures for pre-myopia remain underdeveloped. Although the LAMP Study protocol and low-level red light therapy offer preemptive intervention, both approaches face limitations. The LAMP protocol requires prolonged treatment duration (>3 years),^[22]^ while safety concerns regarding retinal exposure thresholds limit the adoption of light therapy.^[23]^ This therapeutic gap necessitates the identification of earlier biometric or functional biomarkers that precede pre-myopia development.

In this study, the 1-year progression of SE and AL in schoolchildren without myopia at baseline was −0.41 ± 0.32 D and 0.28 ± 0.14 mm, which was consistent with previous studies.^[24]^ The severely depleted hyperopic reserve group (pre-myopia cohort) exhibited accelerated progression (−0.70 ± 0.46 D, 0.40 ± 0.17 mm), while the relative hyperopic insufficiency group progressed at −0.56  ± 0.45 D and 0.31 ± 0.18 mm annually, exceeding rates reported in prior studies.^[25]^ Children who developed myopia within the first year of life showed significantly faster biometric changes. Intervention with DIMS spectacles during the second year attenuated the progression in early converters, aligning with multicenter trial outcomes.^[26]^ Biennial progression rates reached −1.31  ± 0.67 D and 0.74 ± 0.29 mm in the severely depleted group compared with −1.02 ± 0.68 D and 0.59 ± 0.26 mm in the relative insufficiency group. These trajectories mirror the Shanghai Pediatric Cohort findings (−1.00 ± 1.00 D SE and 0.60-mm AL growth over 24 months in 6–8-year-olds),^[27]^ confirming comparable refractive vulnerability between relative hyperopic insufficiency in this study and pre-myopia populations in prior research.

Multivariate logistic regression analysis identified relative hyperopic insufficiency as an independent predictor of 24-month myopia onset, with age-stratified cohorts showing no significant residual age effect. A likely reason for this result is that the definition of hyperopic reserve varies by age. These findings substantiate relative hyperopic insufficiency as a clinically actionable precursor biomarker for pre-myopia. Longitudinal progression patterns revealed a marked acceleration in annualized conversion rates, from 6.0% in year 1 to 35.2% in year 2. This 5.8-fold increase in the conversion hazard during the second surveillance year underscores the critical intervention window between 12- and 24-months post-baseline. Proactive implementation of myopia control strategies during this phase is warranted to disrupt the pathological acceleration trajectories.

The AL/CR ratio is a surrogate indicator of refractive error in cycloplegia and provides critical insights for assessing myopia risk and progression patterns. An AL/CR ratio > 3.0 has been established as a high-risk biomarker for myopic conversion from emmetropia, with elevated ratios demonstrating dose-dependent associations with incident myopia.^[28]^ The severely depleted hyperopic reserve group exhibited AL/CR ratios approaching the critical threshold of 3.0, while the relative hyperopic insufficiency group demonstrated significantly elevated ratios (2.94 ± 0.49) compared with the adequate reserve group (*P* < .001). These findings validate the AL/CR ratio as a discriminative biomarker for identifying children in the relative hyperopic insufficiency phase, a critical premyopic transition period. Elevated AL/CR ratios demonstrate a significant predictive utility for myopia progression in school-aged children, with values exceeding age-specific thresholds serving as reliable indicators of hyperopic reserve depletion. This metric provides a foundational parameter for early intervention protocols, enabling targeted management strategies prior to the onset of clinically defined myopia.

This study has a few methodological constraints that warrant consideration. The limited sample size (n = 206), relatively short follow-up duration (24 months), and suboptimal measurement frequency may have hindered the detection of phases of rapid refractive progression, particularly given the potential latency between biometric changes and clinical myopia onset. Additionally, ocular biometric profiling lacks lens thickness measurements, which is a critical parameter influencing ocular component development.

## 5. Conclusion

Refractive stratification revealed differential myopic progression per millimeter axial elongation in the first year: the severely depleted hyperopic reserve group (−1.75 D/mm) and relative hyperopic insufficiency group (−1.80 D/mm) exhibited significantly greater SE progression than the adequate hyperopic reserve group (−1.40 D/mm) at 12 months (*P* < .001). Notably, the relative hyperopic insufficiency group showed the strongest correlation between axial growth and refractive change, and experienced a marked increase in myopia incidence during the second year of follow-up. Multivariate logistic regression analysis confirmed that relative hyperopic insufficiency was an independent risk factor for myopia onset after 24 months. These findings establish that relative hyperopic insufficiency is a critical precursor biomarker for pre-myopia development. The clinical implementation of proactive preventive measures during the second annual follow-up is strongly recommended to optimize the intervention efficacy.

## Acknowledgments

The authors would like to thank all the participants involved in the study.

## Author contributions

**Data curation:** Chengcheng Han, Haiyan Ma.

**Formal analysis:** Chengcheng Han, Rui Zhou, Haiyan Ma.

**Funding acquisition:** Rui Zhou, Yuyang Wu.

**Methodology:** Yuyang Wu.

**Project administration:** Yuyang Wu.

**Supervision:** Haiyan Ma, Zhiping Zhang.

**Validation:** Qingjie Meng, Zhiping Zhang.

**Visualization:** Qingjie Meng, Zhiping Zhang.

**Writing – original draft:** Chengcheng Han.

**Writing – review & editing:** Chengcheng Han, Zhiping Zhang.
